# Statistical Methods Revisited for Estimating Daily Milk Yields: How Well do They Work?

**DOI:** 10.3389/fgene.2022.943705

**Published:** 2022-08-10

**Authors:** Xiao-Lin Wu, George R. Wiggans, H. Duane Norman, Asha M. Miles, Curtis P. Van Tassell, Ransom L. Baldwin, Javier Burchard, João Dürr

**Affiliations:** ^1^ Council on Dairy Cattle Breeding, Bowie, MD, United States; ^2^ Department of Animal and Dairy Sciences, University of Wisconsin, Madison, WI, United States; ^3^ USDA, Agricultural Research Service, Animal Genomics and Improvement Laboratory, Beltsville, MD, United States

**Keywords:** dairy cattle, days in milk, lactation, exponential growth function, milking interval

## Abstract

Cost-effective milking plans have been adapted to supplement the standard supervised twice-daily monthly testing scheme since the 1960s. Various methods have been proposed to estimate daily milk yields (DMY), focusing on yield correction factors. The present study evaluated the performance of existing statistical methods, including a recently proposed exponential regression model, for estimating DMY using 10-fold cross-validation in Holstein and Jersey cows. The initial approach doubled the morning (AM) or evening (PM) yield as estimated DMY in AM-PM plans, assuming equal 12-h AM and PM milking intervals. However, in reality, AM milking intervals tended to be longer than PM milking intervals. Additive correction factors (ACF) provided additive adjustments beyond twice AM or PM yields. Hence, an ACF model equivalently assumed a fixed regression coefficient or a multiplier of “2.0” for AM or PM yields. Similarly, a linear regression model was viewed as an ACF model, yet it estimated the regression coefficient for a single milk yield from the data. Multiplicative correction factors (MCF) represented daily to partial milk yield ratios. Hence, multiplying a yield from single milking by an appropriate MCF gave a DMY estimate. The exponential regression model was analogous to an exponential growth function with the yield from single milking as the initial state and the rate of change tuned by a linear function of milking interval. In the present study, all the methods had high precision in the estimates, but they differed considerably in biases. Overall, the MCF and linear regression models had smaller squared biases and greater accuracies for estimating DMY than the ACF models. The exponential regression model had the greatest accuracies and smallest squared biases. Model parameters were compared. Discretized milking interval categories led to a loss of accuracy of the estimates. Characterization of ACF and MCF revealed their similarities and dissimilarities and biases aroused by unequal milking intervals. The present study focused on estimating DMY in AM-PM milking plans. Yet, the methods and relevant principles are generally applicable to cows milked more than two times a day.

## Introduction

Accurate milking data are essential for herd management and genetic improvement in dairy cattle. In reality, lactation (305 days) yields are not directly measured, but they are calculated from the test-day yields, either with or without explicitly imputing DMY for non-test dates (VanRaden, 1997; Cole and VanRaden; Cole et al., 2009). For genetic evaluation programs, the standardization of lactation yields is practiced, ensuring that milking records are comparable between cows. The latter goal is to adjust variation due to, for example, the number of milking per day, lactation length, and age and month of calving (McDaniel, 1973; Schutz and Norman, 1994; Norman et al., 1995). Hence, the accuracies of test-day yields form the basis for the accuracies of lactation yields and the following standardization of lactation yields for genetic evaluation programs.

Nevertheless, test-day yields are not directly measured either. In the US, reduced-cost milking plans started to displace the standard supervised twice-daily, monthly testing scheme in the 1960s, motivated by reducing visits by a DHIA supervisor (Puttnam and Gilmore, 1968). Typically, cows are milked two or more times on a test day, but not all these milkings are measured. Porzio (1953) was the first to propose sampling the morning (**AM**) and evening (**PM**) milkings alternately on test days throughout lactation in the mountainous areas of Italy. This was known as the AM-PM milking plan, and the daily yield was taken to be approximately two times the yield of a single milking, assuming equal 12-h intervals for AM and PM milkings. In practice, however, AM and PM milking intervals can be different, and milk secretion rates may vary between day and night. Morning milking intervals tend to be longer than afternoon milking intervals. Hence, AM milk yields are usually higher than PM milk yields (Puttnam and Gilmore, 1970).

Various methods have been proposed to estimate daily milk, fat, and protein yields. The landmark developments date to the 1980s and 1990s, focusing on adjustment criteria in two broad categories, namely, additive (**ACFs**) and multiplicative correction factors (**MCFs**). ACFs provide additive adjustments beyond the two times AM or PM yields as the estimate of daily yields. Everett and Wadell (1970a) showed that the difference between AM and PM yields was a function of milking interval and days in milk (**DIM**). Significant factors affecting differences varied with cattle breeds, which also include lactation months, herd production, age classes, and so on (Everett and Wadell, 1970b). Hence, ACFs are evaluated by the average differences between AM and PM yields milk, say, in AM-PM milking plans, for various milking interval classes (**MICs**), and other categorical variables.

On the other hand, MCFs are ratios of daily yield to yield from a single milking, computed for each MIC. MCFs are also referred to as ratio factors. Multiplying a yield from a single milking by an appropriate ratio factor gives an estimate of daily yield. Various MCF forms have been proposed, yet the statistical interpretations differ (Wu et al., 2022). Shook et al. (1980) described the MCF as reciprocals of the AM or PM portions of daily yields, subject to quadratic smoothing. DeLorenzo and Wiggans (1986) proposed deriving MCF for AM-PM milking plans based on a linear regression model without intercept. They assumed heterogeneous means and variances and fitted separate regression models to each MIC. Wiggans (1986) proposed deriving MCFs for cows milked three times a day by regressing single-to-daily yield ratio on milking interval. Additional predictors such as DIM can be included in the model when applicable. MCF models are statistically challenged by “the ratio problem” because they have a ratio variable (i.e., proportional daily yield) as the dependent variable in the data density (Wiggans, 1986) or the smoothing functions (Shook et al., 1980; DeLorenzo and Wiggans, 1986). Consequences included possible biases in two aspects: omitted variable bias and measurement error bias (Lien et al., 2017). The former bias happens because main model effects are missing if the model is re-arranged by multiplying the denominator variable to both sides of the model equation. The latter bias occurs when there are measurement errors with the denominator variable of the response. Furthermore, the MCF models postulated a rational function between daily milky yield and milking, in which the numerator is 1, and the denominator is a linear function (DeLorenzo and Wiggans, 1986; Wiggans, 1986) or a quadratic function (Shook et al., 1980) of milking interval.

Previous studies almost exclusively assessed the accuracy of estimated daily yield in the same datasets from which the correction factors were derived (Putnam and Gilmore, 1968, 1970; Smith and Person, 1981; Liu et al., 2000). This type of in-sample evaluation essentially reflected more model-fitting accuracy than prediction accuracy. In the present study, our primary goal was to evaluate the performance of existing statistical models, including the recently proposed exponential regression model (Wu et al., 2022), in Holstein and Jersey cattle by cross-validation. Secondary goals included comparing model parameters and characterizing ACF and MCF obtained from various models, relative to the initial approach assuming a fixed multiplicative factor of 2.0 for AM or PM yields. Cross-validation, also referred to as out-of-sample testing, is a model validation technique for assessing how the results of a statistical analysis will generalize to an independent dataset (Stone, 1974; Geisser, 1993). Briefly, one round of cross-validation involves partitioning a sample of data into complementary subsets, performing the analysis on one subset (i.e., training set), and validating the analysis on the other subset (i.e., validation or testing set). To access variability, multiple rounds of cross-validation are performed using different partitions, and the validation results are combined by averaging over the rounds to give an estimate of the model’s predictive performance. Hence, cross-validation combines (averages) measures of fitness in prediction to derive a more accurate estimate of model prediction performance. Because cross-validation is a resampling method that uses different portions of the data to train and test a model across iterations, it also allows inferring the error origins by decomposing an MSE into the variance of the estimate and squared bias. The inverse of the variance provides a measure of precision for the estimates.

## Materials and Methods

### AM-PM Milking Data

Milking records were extracted from the data repositories maintained by the Council for Dairy Cattle Breeding (**CDCB**). The data consisted of 9,218 milking records from 6,533 cows in 27 herds in 11 states, USA, collected from 2006 through 2009 (Table 1). Most milking records consisted of 82.7% Holsteins and 13.1% Jersey (13.1%) cows. The remaining (4.2%) milking records represented multiple breeds, including Ayrshire (0.7%), Brown Swiss (2.4%), Milking Shorthorn (0.01%), Red and White Holstein (0.04%), and unknown breeds (0.87%). Milking records from Holstein and Jersey cows were used in the present study. Data editing excluded records with missing or incomplete values for relevant columns (e.g., AM or PM milking yield, AM or PM milking interval, parity, lactation year or month, days in milk (**DIM**), and herd locations). The final dataset retained 7,544 Holstein milking records from 23 herds and 1,194 Jersey milking records from 9 herds. Approximately, one-third of records (30.6–39.9%) represented the first parity cows and two-thirds (59.4–69.4%) were the second parity cows in the two breeds. Milking records collected from parity 3 and greater were rate (0–10.7%).

**TABLE 1:** Number (n) and percentage (%n) of milking records by parities, lactation years, and states in the Holstein and Jersey cattle, respectively.

Variable		Holstein		Jersey	
		n	%n	n	%n
Parity	1	3,006	39.9	366	30.6
	2	4,482	59.4	831	69.4
	3+	56	0.70	0	0
SUM		7,544	100	1,197	100
Year	2006	153	2.00	434	36.3
	2007	338	4.50	0	0
	2008	7,000	92.8	360	30.1
	2009	53	0.70	403	33.7
SUM		7,544	100	1,197	100
State	Vermont	1,738	23.0	4	0.30
	New York	361	4.80	182	15.2
	Pennsylvania	1,224	16.2	333	27.8
	Indiana	375	5.00	206	17.2
	Minnesota	338	4.50	0	0
	Iowa	153	2.00	434	36.3
	Delaware	511	6.80	2	0.20
	Maryland	900	11.9	0	0
	West Virginia	252	3.30	0	0
	Georgia	945	12.5	36	3.00
	Florida	747	9.90	0	0
SUM		7,544	100	1,197	100

### Statistical Methods

#### Model 0 (M0): Doubling AM or PM Milking Yield

The initial AM-PM milking plan alternately sampled AM or PM milking on a test day throughout lactation, and the daily yield was obtained by doubling single milk weighed on each test day (Porzio, 1953). That is,

$${\hat{y}}_{ij}=2x_{ij},$$ (1)

where $x_{ij}$ is the known AM ( j=1 ) or PM ( j=2 ) yield for cow *i*, and ${\hat{y}}_{ij}$ is an estimated DMY. Doubling AM or PM milk yield is equivalent to assuming a fixed multiplicative correction factor for all cows, assuming equal (12–12 h) AM and PM milking intervals.

#### Model 1 (M1): ACF Model With Discrete Variables

Additive correction factors are evaluated by the expected values of the differences between AM and PM yields, computed locally for each MIC, coupled with other categorical variables such as lactation months (Everett and Wadell, 1970b). For example, let ${\text{z}}_{ijkl}$ be the difference between AM and PM milk yield for cow *i*, pertaining to MIC *k* and lactation month (LM) *l*. Assume that the yield from milking *j* is measured. The data model accounting for variations due to MIC and LM is the following:

$${\text{z}}_{ijkl}=\mu_{j}+MIC_{k}+LM_{l}+{\left(MIC\;*\;LM\right)}_{kl}+\epsilon_{ijkl},$$ (2)

where $\mu_{j}$ is the overall mean for milking *j*, $MIC_{k}$ and $LM_{l}$ are the main effects for MIC *k* and LM *l*, respectively, ${\left(MIC\;*\;LM\right)}_{kl}$ is the interaction effect, and $\epsilon_{ijkl}$ is an error term. Then, ACF (denoted by $\Delta_{jkl}$ ) are computed by

$$\Delta_{jkl}=E\left(z_{ijkl}\right),$$

$$\approx {\hat{\mu }}_{j}+{\hat{MIC}}_{j}+{\hat{LM}}_{k}+{\hat{\left(MIC*LM\right)}}_{jk}.$$ (3)

Given the computed ACF and a single milk yield that has been measured for cow *i* (denoted by $x_{ijkl}$ ), the estimated daily milk yield (**DMY**, denoted by ${\hat{y}}_{ijkl}$ ) is obtained as follows:

$${\hat{y}}_{ijkl}=\Delta_{jkl}+2x_{ijkl}.$$ (4)

In the aforementioned equation, we see that an ACF model is equivalent to a regression model assuming a fixed regression coefficient (2.0) for AM or PM yield. ACF models can be fit on AM or PM milk yields separately or jointly.

#### Model 2 (M2A,B): ACF Model With Continuous Variables

An ACF model can also be fitted with continuous variables for milking interval (denoted by $t_{ij}$ ) and DIM (denoted by $d_{ij}$ ), assuming heterogeneous intercepts and common slopes for milking interval and DIM, respectively, as follows.

$$z_{ij}=\alpha_{j}+\beta t_{ij}+\gamma \left(d_{ij}-d_{0}\right)+\epsilon_{ij},$$ (5)

where $z_{ij}$ is the difference between milking j and the other milking for cow *i*, $\alpha_{j}$ is the intercept for milking *j*, β and γ are the common regression coefficients for milking interval and DIM, respectively, $d_{0}$ is an arbitrary constant value for DIM, say, $d_{0}=158$ , and $\epsilon_{ij}$ is an error. Here, DIM is used as a continuous variable, instead of the categorical LM.

Given the estimated model parameters, DMY is estimated by

$${\hat{y}}_{ij}={\hat{\alpha }}_{j}+\hat{\beta }t_{ij}+\hat{\gamma }\left(d_{ij}-d_{0}\right)+2x_{ij},$$ (6)

where $x_{ij}$ is the measured yield from milking *j* for cow *i*. By this approach, the model is referred to as M2A. Alternatively, ACF are computed for discretized MIC, say MIC *k* of milking *j* (denoted by $\Delta_{j}^{\left(k\right)}$ ):

$$\Delta_{j}^{\left(k\right)}={\hat{\alpha }}_{j}+\hat{\beta }{\bar{t}}_{j}^{\left(k\right)},$$ (7)

where ${\bar{t}}_{j}^{\left(k\right)}$ is a midpoint of MIC *k*. Here, we used superscript “(k)” to pinpoint discretized MIC, which distinguishes from a subscript *k* for a categorical variable for MIC in the model. This notation is used throughout this report. Then, DMY is estimated by

$${\hat{y}}_{ij}=\Delta_{j}^{\left(k\right)}+\hat{\gamma }\left(d_{ij}-d_{0}\right)+2x_{ij}.$$ (8)

With the latter approach (denoted by M2B), DMY is estimated through the ACF.

#### Model 3 (M3A,B): Linear Regression With Linear Milking Interval and DIM

The linear model approach treats DMY as the response variable. Let $y_{ij}$ be a daily yield for cow *i* on milking *j*, $x_{ij}$ be a yield from a single milking from milking *j*, $t_{ij}$ be the milking interval time, and $d_{ij}$ be the responding DIM for the test date. Then, the linear regression model accounting for the aforementioned variables is the following:

$$y_{ij}=\alpha_{j}+\beta t_{ij}+\gamma \left(d_{ij}-d_{0}\right)+bx_{ij}+\epsilon_{ij}.$$ (9)

In (9), $\alpha_{j}$ is an overall mean specific to milking *j*, β , γ , and b are common regression coefficients for milking interval, DIM, and single milk (AM or PM) yield, respectively, and $\epsilon_{ij}$ is an error.

Linear regression also offers two methods of estimating DMY. First, DMY for a cow can be estimated directly given the estimated model parameters in (9), as follows:

$${\hat{y}}_{ij}={\hat{\alpha }}_{j}+\hat{\beta }t_{ij}+\hat{\gamma }\left(d_{ij}-d_{0}\right)+\hat{b}x_{ij}.$$ (10)

The aforementioned equation is referred to as the model M3A. Second, ACF can be computed on discretized MIC, following the same formula as (7), and then DMY are estimated by the following (denoted by M3B):

$${\hat{y}}_{ij}={\text{Δ}}_{j}^{\left(k\right)}+\hat{\gamma }\left(d_{ij}-d_{0}\right)+\hat{b}x_{ij}.$$ (11)

#### Model 4 (M4): Linear Regression With Linear and Quadratic Milking Interval and DIM

Linear regression models can be defined with varying complexity (Liu et al., 2000; Schaeffer et al., 2000). In the present study, we also evaluated a linear regression model with linear and quadratic variables for milking interval and DIM:

$$y_{ij}=\alpha_{j}+\beta_{1}t_{ij}+\beta_{2}t_{ij}^{2}+\gamma_{1}\left(d_{ij}-d_{0}\right)+r_{2}{\left(d_{ij}-d_{0}\right)}^{2}+bx_{ij}+\epsilon_{ij}.$$ (12)

Given the estimated model parameters, DMY is estimated directly as follows:

$${\hat{y}}_{ij}={\hat{\alpha }}_{j}+{\hat{\beta }}_{1}t_{ij}+{\hat{\beta }}_{2}t_{ij}^{2}+{\hat{\gamma }}_{1}\left(d_{ij}-d_{0}\right)+{\hat{\gamma }}_{2}{\left(d_{ij}-d_{0}\right)}^{2}+\hat{b}x_{ij}.$$ (13)

MCF could be derived similar to M2B, yet considering the quadratic terms, but they were not evaluated in the present study.

#### Model 5 (M5): The 1980 Shook-Jensen-Dickimson MCF model

Shook et al. (1980) described MCF by the inverse of AM or PM proportion of daily milk yield. For example, MCF given PM yields are formulated as follows:

$$F_{jk}=\frac{AMP_{jk}+PMP_{jk}}{PMP_{jk}},$$ (14)

where j=2 (PM), and $AMP_{k}$ and $PMP_{k}$ stand for bulk AM and PM yields, respectively, for MIC *k* in a population. Shook et al. (1980) employed a quadratic regression of the PM portion of DMY on MIC midpoints, and smoothed estimates of MCF were obtained as follows:

$$F_{jk}=\frac{1}{{\hat{\alpha }}_{j}+{\hat{\beta }}_{j1}{\bar{t}}_{jk}+{\hat{\beta }}_{j2}{\bar{t}}_{jk}^{2}}.$$ (15)

In the aforementioned equation, ${\hat{\alpha }}_{j}$ , ${\hat{\beta }}_{j1}$ , and ${\hat{\beta }}_{j2}$ are the estimated intercept and regression coefficients in the quadratic smoothing function, and ${\bar{t}}_{jk}$ is the midpoint of MIC *k* for milking *j* . The quadratic smoothing also provided estimates for MIC with no or insufficient milking records.

Given the estimated PM MCF, the AM MCF can be computed indirectly (Shook et al., 1980), but this approach was not taken in the present study. Instead, we computed AM and PM MCF directly from the AM or PM milking data. Similar to (14), MCF given AM yields are formulated to be the inverses of the AM portion of daily yield, computed for each AM MIC ( j=1) :

$$F_{jk}=\frac{AMP_{jk}+PMP_{jk}}{AMP_{jk}}.$$ (16)

Given the MCF ( $F_{jk}$ ) and the yield from single milking *j* for an animal, say *i*, measured on MIC *k* ( $x_{ijk}$ ), DMY for this animal is estimated by

$${\hat{y}}_{ijk}=F_{jk}x_{ijk}.$$ (17)

#### Model 6 (M6): The 1986 DeLorenzo and Wiggans MCF model

DeLorenzo and Wiggans (1986) derived MCF for cows milked twice a day based on a linear regression without intercept. They assumed heterogeneous means and variances and fitted separate linear regression models for different MIC.

$$y_{ijk}=b_{jk}x_{ijk}+\gamma_{jk}\left(d_{ijk}-d_{0}\right)+\epsilon_{ijk}$$ (18)

In (18), $b_{jk}$ is the regression coefficient for single milk yield, and $\gamma_{jk}$ is the regression coefficient of DIM. Here, the regression coefficient, $b_{jk}$ , coincides with the multiplicative correction factor, as defined by Shook et al. (1980) derived for MIC *k* of milking *j*, assuming $E\left(d_{ijk}-d_{0}\right)=0$ . DeLorenzo and Wiggans (1986) employed a linear regression smoothing for the reciprocals of computed AM and PM factors, respectively:

$$F_{j}^{\left(k\right)}=\frac{1}{{\hat{\alpha }}_{j}+{\hat{\beta }}_{j}{\bar{t}}_{jk}}.$$ (19)

Given the computed MCF, DMY is estimated by

$${\hat{y}}_{ij}^{\left(k\right)}=F_{ij}^{\left(k\right)}x_{ij}^{\left(k\right)}+{\hat{\gamma }}_{jk}\left(d_{ij}^{\left(k\right)}-d_{0}^{\left(k\right)}\right).$$ (20)

#### Model 7 (M7A,B): The 1986 Wiggans MCF model

Wiggans (1986) proposed to derive MCF for cows milked three times a day by modeling the single-to-daily milk yield ratio as a linear function of milking interval and DIM when applicable:

$$\frac{x_{ij}}{y_{ij}}=\alpha_{j}+\beta t_{ij}+\gamma \left(d_{ij}-d_{0}\right)+\epsilon_{ij}.$$ (21)

The aforementioned model also applies to cows milked more than three times and, arguably, it applies to cows milked twice a day. In the latter case, however, the model is subject to the violation of linearity with a longer milking interval (Schmidt, 1960). In the present study, DMY is estimated directly based on the estimated model parameters from (21) or through computed MCF according to Wiggans (1986). In the former case (denoted by M7A), DMYs are computed directly given the estimated model parameters, as follows:

$${\hat{y}}_{ij}=\frac{x_{ij}}{{\hat{\alpha }}_{j}+\hat{\beta }t_{ij}+\hat{\gamma }\left(d_{ij}-d_{0}\right)}.$$ (22)

In the latter case (denoted by M7B), MCF are obtained by locally taking the expected value on both sides of Equation 21, assuming $E\left(\gamma \left(d_{ij}^{\left(k\right)}-d_{0}^{\left(k\right)}\right)\right)=0$ and $E\left(\epsilon_{ij}^{\left(k\right)}\right)=0$ . In other words,

$$F_{j}^{\left(k\right)}=\frac{1}{\alpha_{j}+\beta {\bar{t}}_{j}^{\left(k\right)}}.$$ (23)

Similarly, by taking the first-order Taylor series approximation of (21), that is, $E\left(\frac{x_{ij}}{y_{ij}}\right)\approx \frac{E\left(x_{ij}\right)}{E\left(y_{ij}\right)}$ , and assuming $E\left(\epsilon_{ij}\right)=0$ , DMY is estimated using the same formula as (20).

#### Model 8 (M8A,B): Exponential Regression Model

Considering milking interval and days in milk, the exponential regression model for estimating DMY takes the following form (Wu et al., 2022):

$$y_{ij}=x_{ij}^{b}\;e^{\left(\alpha_{j}+\beta t_{ij}+\gamma \left(d_{ij}-d_{0}\right)+\epsilon_{ij}\right)}.$$ (24)

By noting e≈2.718, the exponential function is analogous to an exponential growth (or decay) function, given its initial value $y_{0}=x_{ij}^{b}$ :

$$y=y_{0}{\left(1+r\right)}^{t^{*}},$$ (25)

where r=1.718 is the rate of change, tuned by a time function, $E\left(t^{*}\right)=\alpha_{j}+\beta t_{ij}+\gamma \left(d_{ij}-d_{0}\right)$ , as a linear function of milking interval and days in milk, and $y_{0}=x^{b}$ is the initial state. Here, y has an exponential growth when $t^{*}>0$ , or an exponential decay when $t^{*}<0$ .

The model parameters can be estimated by taking the following logarithm transformation:

$$log\left(y_{ij}\right)=\alpha_{j}+\beta t_{ij}+\gamma \left(d_{ij}-d_{0}\right)+blog\left(x_{ij}\right)+\epsilon_{ij}.$$ (26)

As a direct approach. DMY is estimated, given the model parameter estimates ( $\hat{b}$ , ${\hat{\alpha }}_{j}$ , $\hat{\beta }$ , and $\hat{\gamma }$ ) (denoted as the model M8A), assuming $E\left(\epsilon_{ij}\right)=0$ . In other words,

$${\hat{y}}_{ij}=x_{ij}^{\hat{b}}\;e^{\left({\hat{\alpha }}_{j}+\hat{\beta }t_{ij}+\hat{\gamma }\left(d_{ij}-d_{0}\right)\right)}.$$ (27)

Alternatively, MCF is computed locally for discretized MIC (Wu et al., 2022):

$$F_{j}^{\left(k\right)}=E{\left(x_{ij}^{\left(k\right)}\right)}^{b-1}\rho_{j}^{\left(k\right)}e^{\left({\hat{\alpha }}_{j}+\hat{\beta }{\bar{t}}_{j}^{\left(k\right)}\right)},$$ (28)

where $\rho_{j}^{\left(k\right)}=e^{\frac{1}{2}\left(V\left(y_{ij}^{\left(k\right)}\right)E{\left(y_{ij}^{\left(k\right)}\right)}^{-2}-bV\left(x_{ij}^{\left(k\right)}\right)E{\left(x_{ij}^{\left(k\right)}\right)}^{-2}\right)}$ , and $E\left(y_{ij}^{\left(k\right)}\right)={\bar{y}}_{j}^{\left(k\right)}$ and $E\left(x_{ij}^{\left(k\right)}\right)={\bar{x}}_{j}^{\left(k\right)}$ are the corresponding means for daily yield and AM (or PM) yield. Then, DMY is estimated by

$${\hat{y}}_{ij}^{\left(k\right)}=F_{j}^{\left(k\right)}x_{ij}^{\left(k\right)}\times e^{\hat{\gamma }\left(d_{ij}^{\left(k\right)}-d_{0}^{\left(k\right)}\right)}.$$ (29)

The logarithm linear regression also suggests that ACF can be computed for estimating $log\left(y_{ij}\right)$ , and then, DMY in its original scale can be computed conveniently by taking an exponential transformation. The option for computing ACF based on the exponential regression model was not evaluated in the study.

### Cross-Validation of Accuracy

The performance of eight selected models and two strategies (Table 2) was evaluated for estimating DMY in the Holstein and Jersey milking datasets. The eight models included two ACF models, one with discrete MIC (M1) and the other with a continuous variable for milking interval (M2), a linear regression model M3 and M4, and three MCF models (M5, M6, and M7), according to Shook et al. (1980), DeLorenzo and Wiggans (1986), and Wiggans (1986), respectively, and the exponential regression model (M8), with doubling AM or PM (M0) as the benchmark model for comparison. For the two strategies, a model labeled “A” (M2A, M3A, M7A, and M8A) estimated DMY directly, given the estimates of model parameters, whereas a model labeled “B” (M2B, M3B, M7B, and M8B) estimated DMY indirectly *via* the computed ACF or MCF. Accuracy and decomposed mean squared errors (MSE) were evaluated for each model or model–strategy combination by cross-validation. Briefly, each dataset was divided into 10 approximately equal subsets. Then, nine subsets were pooled for training, and the remaining subset was used for testing the accuracy. The cross-validation process rotated 10 times, with each subset used for testing once and only once. To facilitate inference of the variance of the estimates, cross-validations were replicated 30 times, each with randomly selected subsets of data samples for training and testing.

**TABLE 2:** Statistical methods and correction factors used in the present study^a^ ^,^ ^b^ ^,^ ^c^.

Model	Equation	Additive ( Δ ) or ratio (F) Factor
M0	$y_{ij}=2x_{ij}$	F≡2
M1	$y_{ijkl}=\mu_{j}+MIC_{k}:LM_{l}+2x_{ijkl}+\epsilon_{ijkl}$	$\Delta_{jk}={\hat{\mu }}_{j}+\hat{MIC_{j}:LM_{k}}$
M2A	$y_{ij}=\alpha_{j}+\beta t_{ij}+\gamma \left(d_{ij}-d_{0}\right)+2x_{ij}+\epsilon_{ij}$	---
M2B	$y_{ij}=\alpha_{j}+\beta t_{ij}+\gamma \left(d_{ij}-d_{0}\right)+2x_{ij}+\epsilon_{ij}$	$\Delta_{j}^{\left(k\right)}={\hat{\alpha }}_{j}+\hat{\beta }{\bar{t}}_{j}^{\left(k\right)}+\hat{\gamma }E\left(d_{ij}^{\left(k\right)}-d_{0}^{\left(k\right)}\right)$
M3A	$y_{ij}=\alpha_{j}+\beta t_{ij}+\gamma \left(d_{ij}-d_{0}\right)+bx_{ij}+\epsilon_{ij}$	---
M3B	$y_{ij}=\alpha_{j}+\beta t_{ij}+\gamma \left(d_{ij}-d_{0}\right)+bx_{ij}+\epsilon_{ij}$	$\Delta_{j}^{\left(k\right)}={\hat{\alpha }}_{j}+\hat{\beta }{\bar{t}}_{j}^{\left(k\right)}+\hat{\gamma }E\left(d_{ij}^{\left(k\right)}-d_{0}^{\left(k\right)}\right)$
M4	$y_{ij}=\alpha_{j}+\beta_{1}t_{ij}+\gamma_{1}\left(d_{ij}-d_{0}\right)+\beta_{2}t_{ij}^{2}+\gamma_{2}{\left(d_{ij}-d_{0}\right)}^{2}+bx_{ij}+\epsilon_{ij}-$	---
M5	$\frac{\sum_{i}x_{ij}^{\left(k\right)}}{\sum_{i}y_{ij}^{\left(k\right)}}=\alpha_{j}+\beta_{j1}{\bar{t}}_{j}^{\left(k\right)}+\beta_{j2}{\left({\bar{t}}_{j}^{\left(k\right)}\right)}^{2}+\epsilon_{j}$	$F_{j}^{\left(k\right)}=\frac{1}{{\hat{\alpha }}_{j}+{\hat{\beta }}_{j1}{\bar{t}}_{j}^{\left(k\right)}+{\hat{\beta }}_{j2}{\left({\bar{t}}_{j}^{\left(k\right)}\right)}^{2}}$
M6	$y_{ijk}=b_{jk}x_{ijk}+\varepsilon_{ijk};{\left({\hat{b}}_{j}^{\left(k\right)}\right)}^{-1}=\alpha_{j}+\beta_{j}{\bar{t}}_{j}^{\left(k\right)}+\epsilon_{j}$	$F_{j}^{\left(k\right)}=\frac{1}{\hat{\alpha }+\hat{\beta }{\bar{t}}_{j}^{\left(k\right)}}$
M7A	$\frac{x_{ij}}{y_{ij}}=\alpha_{j}+\beta t_{ij}+\gamma \left(d_{ij}-d_{0}\right)+\epsilon_{ij}$	
M7B	$\frac{x_{ij}}{y_{ij}}=\alpha_{j}+\beta t_{ij}+\gamma \left(d_{ij}-d_{0}\right)+\epsilon_{ij}$	$F_{j}^{\left(k\right)}=\frac{1}{\hat{\alpha }+\hat{\beta }{\bar{t}}_{j}^{\left(k\right)}}$
M8A	$y_{ij}=x_{ij}^{b}e^{\left(\alpha_{j}+\beta t_{ij}+\gamma \left(d_{ij}-d_{0}\right)+\epsilon_{ij}\right)}$	---
M8B	$y_{ij}=x_{ij}^{b}e^{\left(\alpha_{j}+\beta t_{ij}+\gamma \left(d_{ij}-d_{0}\right)+\epsilon_{ij}\right)}$	$F_{j}^{\left(k\right)}=\rho^{*}e^{{\hat{\alpha }}_{j}+\hat{\beta }{\bar{t}}_{j}^{\left(k\right)}}$

a M0 = daily milk yield (DMY) estimated by doubling morning (AM) or evening (PM) milk yield; M1 = additive correction factor (ACF) model with categorical milking interval classes (MIC) and lactation months; M2A = ACF model with continuous variables for milking interval and days in milk (DIM); M2B = M2A with ACF computed on discretized MIC; M3A = linear regression of daily milk yield on milking interval and DIM; M3B = M3A with ACF computed on discretized MIC; M4 = M3A with quadratic terms for milking interval and DIM; M5 = multiplicative correction factor (MCF) model according to Shook et al. (1980); M6 = MCF model according to DeLorenzo and Wiggans (1986); M7A = linear regression of AM or PM proportion of DMY on milking interval and DIM (Wiggans, 1986); M7B = M7A with MCF computed for discretized MIC (Wiggans, 1986); M8A = exponential regression model (Wu et al., 2022); M8B = M8A with MCF computed on discretized MIC.

b ${\bar{t}}_{j}^{\left(k\right)}$ = midpoint of milking interval *k* of milking *j*, for j=1 (AM milking) or 2 (PM milking): $\rho^{*}=e^{\frac{1}{2}\left(V\left(y_{ij}^{\left(k\right)}\right)E{\left(y_{ij}^{\left(k\right)}\right)}^{-2}-bV\left(x_{ij}^{\left(k\right)}\right)E{\left(x_{ij}^{\left(k\right)}\right)}^{-2}\right)}\times \frac{E{\left(x_{ij}^{\left(k\right)}\right)}^{b}}{E\left(x_{ij}^{\left(k\right)}\right)}$ .

c --- = computing yield correction factors is not required.

The correlation between the estimate and actual DMY and the following *R* ^2^ accuracy:

$$R^{2}=\frac{\sigma^{2}}{\sigma^{2}+MSE}.$$ (30)

Here, $\sigma^{2}$ was the true phenotype of DMY, assuming actual DMY was obtained without measurement error, and MSE was mean squared error. The *R* ^2^ accuracy was calculated per cross-validation population-wise or per individual animal. In the former case, MSE were obtained as the population parameter and the *R* ^2^ accuracy was calculated for each cross-validation replicate. Then, the mean and standard deviation (also referred to as the standard error) of the *R* ^2^ accuracy and correlation estimates were obtained across the 30 cross-validation replicates. In the latter case, the MSE was calculated as the average across the 30 replicates for each animal, and individual *R* ^2^ accuracy was calculated according to Equation 30 per animal.

To infer the origin of errors, the mean squared error (MSE) of DMY estimates from the 10-fold cross-validation was decomposed into the variance ( $Var\left({\hat{y}}_{i}\right)$ ) and the squared bias ( $Bias^{2}\left({\hat{y}}_{i}\right)$ ), as follows:

$$MSE=\frac{1}{n\times m}\overset{n}{\underset{i=1}{\sum }}\overset{m}{\underset{r=1}{\sum }}{\left({\hat{y}}_{ir}-y_{i}\right)}^{2}=\frac{1}{n\times m}\overset{n}{\underset{i=1}{\sum }}\overset{m}{\underset{r=1}{\sum }}{\left({\hat{y}}_{ir}-{\bar{\hat{y}}}_{i}\right)}^{2}+\frac{1}{n}\overset{n}{\underset{i=1}{\sum }}{\left({\bar{\hat{y}}}_{i}-y_{i}\right)}^{2}=Var\left({\hat{y}}_{i}\right)+Bias^{2}\left({\hat{y}}_{i}\right).$$ (31)

In the aforementioned equation, $Var\left({\hat{y}}_{i}\right)=\frac{1}{n\times m}\overset{n}{\underset{i=1}{\sum }}\overset{m}{\underset{r=1}{\sum }}{\left({\hat{y}}_{ir}-{\bar{\hat{y}}}_{i}\right)}^{2}$ and $Bias^{2}\left({\hat{y}}_{i}\right)=\frac{1}{N}\overset{N}{\underset{i=1}{\sum }}{\left({\bar{\hat{y}}}_{i}-y_{i}\right)}^{2}$ , where *n* is the number of animals, *m* is the number of replicates, $y_{i}$ was a true DMY for cow *i*, ${\hat{y}}_{ir}$ was an estimate of daily milking yield from the *rth* replicate, and ${\bar{\hat{y}}}_{i}$ was the average of the estimated DMY across the 30 replicates.

### Cubic Smoothing Splines

Cubic smoothing splines of the individual *R* ^2^ accuracies and actual daily milk yields, respectively, were also fitted to provide approximations with weaker assumptions for relevant comparisons. Statistically, smoothing splines are function estimates (denoted by $\hat{f}\left(x\right)$ ) obtained from a set of noisy observations $y_{i}$ of the target $f\left(x_{i}\right)$ , which balance a measure of goodness of fit of $\hat{f}\left(x\right)$ to $y_{i}$ with a derivative-based measurement of the smoothness of $\hat{f}\left(x\right)$ (Craven and Wahba., 1979). A *k*th order spline is a piecewise polynomial function of degree k, which is continuous and has continuous derivatives of orders 1,. . ., *k* − 1, at its knot points.

Let $\left\{x_{i},y_{i};i=1,\ldots ,n\right\}$ be a set of observations governed by the relation $y_{i}=f\left(x_{i}\right)+\epsilon_{i}$ . The cubic smoothing spline estimate $\hat{f}$ of the function f is defined to be the minimizer of the following, over the class of twice differentiable functions,

$$\sum_{i=1}^{n}{\left(y_{i}-\hat{f}\left(x_{i}\right)\right)}^{2}+\lambda \int {\hat{f}}^{\prime \prime }{\left(x\right)}^{2}dx.$$ (32)

In the aforementioned equation, λ≥0 is a smoothing parameter, controlling the trade-off between fidelity to the data and roughness of the function estimate. This is often estimated by generalized cross-validation or by restricted marginal likelihood (REML) which exploits the link between spline smoothing and Bayesian estimation (because the smoothing penalty can be viewed as being induced by a prior on the f). The integral is often evaluated over the whole real line, although it is also possible to restrict the range to that of $x_{i}$ . As λ→0 (no smoothing), the smoothing spline converges to the interpolating spline. As λ→∞ (infinite smoothing), the roughness penalty becomes paramount and the estimate converges to a linear least squares estimate. The roughness penalty based on the second derivative is the most common in the modern statistics literature, although the method can easily be adapted to penalties based on other derivatives. The penalized sum of squares smoothing objective can be replaced by a *penalized likelihood* objective in which the sum of squares terms is replaced by another log-likelihood-based measure of fidelity to the data. The sum of squares term corresponds to penalized likelihood with a Gaussian assumption on the $\epsilon_{i}$ .

## Results and Discussion

### Summary of Milking Data for Holstein and Jersey Cows

**FIGURE 1:**
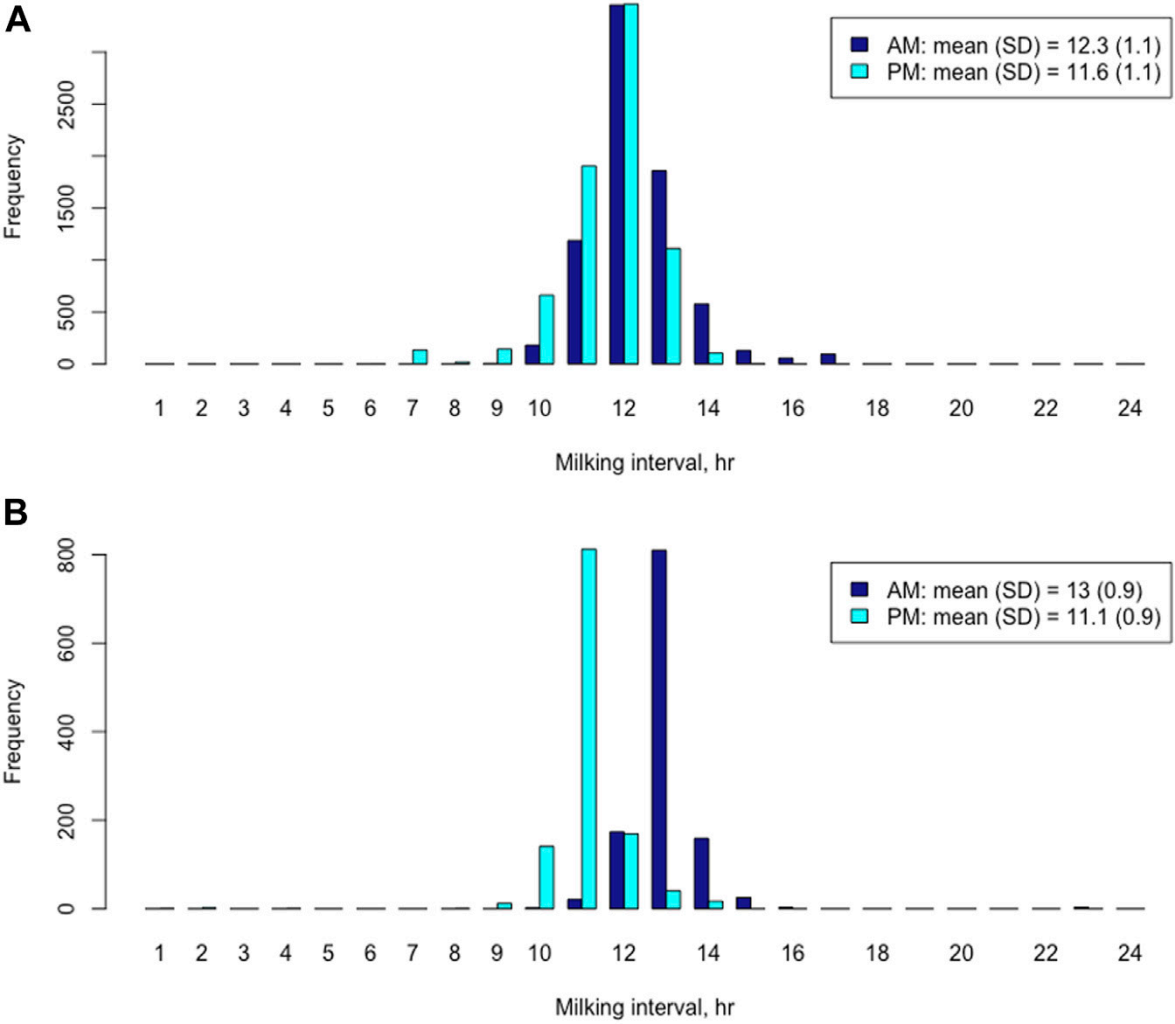
Distributions of morning (AM) and evening (PM) milking interval time in Holstein cows **(A)** and Jersey cows **(B)**, respectively.

In the Holstein cows, the mean and median of AM milking intervals were 12.3 h and 12.1 h, respectively, whereas the mean and median of PM milking intervals were 11.6 h and 11.9 h, respectively. The AM milking intervals had a wider range (5.6–23.67 h) than the PM milking intervals (5.0–18.4 h) (Figure 1A). A paired *t*-test showed that the mean AM milking interval was significantly longer than the mean PM milking interval in the Holsteins cows (t = 27.3, *p* < 2.2e-16). The mean difference between AM and PM milking intervals was 0.688 h, with a 95% confidential interval between 0.639 h and 0.738 h. Similarly, the mean and median of AM milking intervals in the Jersey cows were 13.0 h and 12.9 h, respectively. The mean and median of PM milking intervals were 11.1 h and 11.0 h, respectively. The AM milking interval was significantly longer than the PM milking interval based on a paired *t*-test (t = 44.2; *p* < 2.2e-16). The mean difference between AM and PM milking interval in the Jersey cows was 1.87 h, with a 95% confidential interval between 1.79 h and 1.95 h. The AM milking interval range (9.6–23.5 h) was also larger than the PM milking interval range (1.4–14.3 h) in Jersey cows (Figure 1B). The distribution of AM and PM milking intervals was approximately symmetric and bell shaped in the Holstein and Jersey cows, respectively (Figure 1).

**FIGURE 2:**
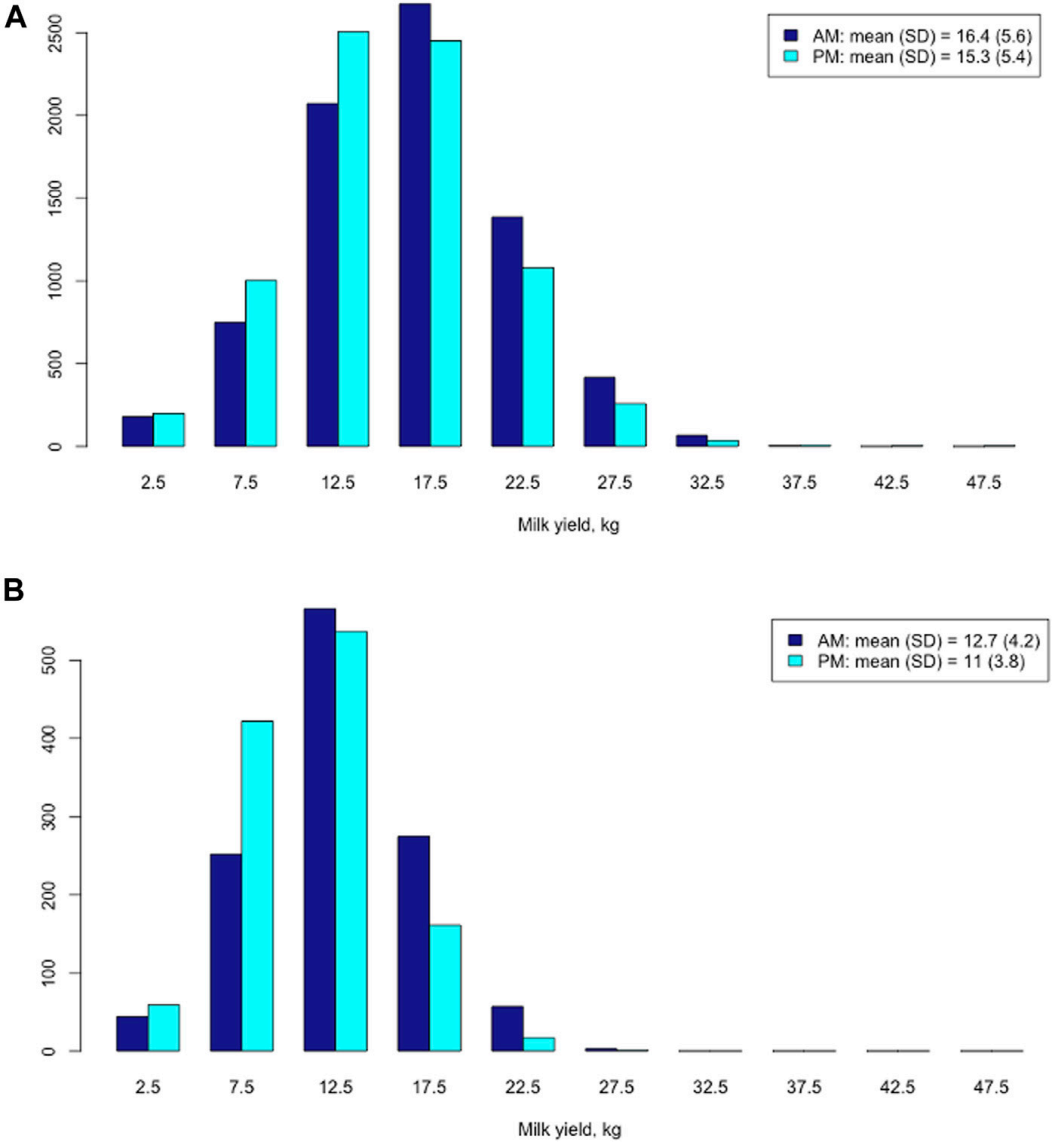
Distribution of morning (AM) and evening (PM) milk yields in Holstein cows **(A)** and Jersey cows **(B)**, respectively.

Longer AM milking intervals led to greater average AM milk yields (Figure 2). In the Holstein cows, the mean AM milk yield (16.4 kg) was significantly larger than the average PM milk yield (15.3 kg) (t = 23.5; *p* < 2.2e-16) (Figure 2A). The mean difference between AM and PM milk yield was 2.49 kg, with a 95% confidential interval between 2.29 and 2.70 kg, in the Holstein cows. Similarly, the mean AM milk yield (12.7 kg) was significantly larger than the average PM milk yield (11.0 kg) (t = 22.2; *p* < 2.2e-16) in the Jersey cows (Figure 2B). The mean difference between AM and PM milk yield was 3.87 kg, with a 95% confidential interval between 3.53 and 4.21 kg, in the Jersey cows.

### Comparing Decomposed Mean Squared Errors and Accuracies

Accuracy and precision are two primary measures of observational or estimation errors. For estimating DMY, accuracy tells how close an estimated DMY is to the actual value, whereas precision shows how well the estimates agree with each other. Precision was measured by the inverse of the variance of DMY estimates. The smaller the variance, the greater the precision. Decomposed MSE were shown in Table 3. All the methods had close to zero variances for the DMY estimates, meaning they all had high precision of the estimated DMY. The variance of DMY estimates was not greater than 0.003 in Holstein cows and less than 0.03 in Jersey cows. The MSE were dominated by the portion of squared bias in Holstein and Jersey cows. Model M0 (doubling AM or PM milk yields) had the largest squared biases and the largest MSE, which were more than two times their counterparts for all the other models in Holstein and Jersey cows. Comparably speaking, the ACF models had larger squared biases and MSE than the MCF and linear regression models. The exponential regression model (M8A) had the smallest squared biases and the smallest MSE. Not including the model M0, the root MSE was between 3.18 and 3.38 kg in the Holstein cows and between 2.46 and 2.63 kg in the Jersey cows. The root MSE roughly agreed with two or three Schutz and Norman (2011), who reported a range of root MSE between 2.07 and 2.85 kg for cows milked twice a day. Higher root MSE for estimating DMY were reported in cows milked times a day (Schutz et al., 2008). It is worth mentioning that we used a 10-fold cross-validation, whereas Schutz and Norman (2011) employed an in-sample evaluation. Often, cross-validations tend to report higher errors than in-sample evaluations when applied to the same dataset. In-sample errors are the errors we get on the same data we used to train the prediction model, which tends to be optimistic, compared to the errors we would get from a new sample. The latter is referred to as out-of-sample errors. The reason is overfitting with in-sample evaluation (Harkins and Douglas, 2004). Overfitting occurs when the trained predictive model becomes sensitive to the noise in the sample. As a result, the function will perform well on the training set but not perform well on new data. The more overfitting occurs, the worse the predictive model will generalize to new data. When we get a new dataset, there will be different noises, so the accuracy will go down to some extent. Hence, in-sample errors are always less than out-of-sample errors, which leads to overestimated accuracy. Yet, the fact is, once we build a model on a sample of data that we have collected, we might want to test the realistic expectation of the predictive model as to how well it will perform on new data.

**TABLE 3:** Decomposed mean squared error, *R* ^2^ accuracy, and correlation between estimated and actual daily milk yield obtained from 10-fold cross-validation ^a^ ^,^ ^b^ ^,^ ^c^.

Method	Holstein					Jersey					
	Varb	Bias^2^	MSE	Acc	Cor	Varb	Bias^2^	MSE	Acc	Cor	
M0	0	22.8	22.8	0.821 (0)	0.927 (0)	0.000	14.54	14.54	0.798 (0)	0.948 (0)
M1	0.003	11.3	11.3	0.902 (<0.001)	0.951 (<0.001)	0.012	6.718	6.730	0.895 (<0.001)	0.952 (0.001)	
M2A	<0.001	11.3	11.3	0.902 (<0.001)	0.951 (<0.001)	0.002	6.910	6.912	0.892 (<0.001)	0.952 (<0.001)	
M2B	<0.001	11.4	11.4	0.902 (<0.001)	0.951 (<0.001)	0.002	6.746	6.748	0.895 (<0.001)	0.952 (<0.001)	
M3A	<0.001	10.3	10.3	0.910 (<0.001)	0.951 (<0.001)	0.002	6.078	6.080	0.904 (<0.001)	0.953 (<0.001)	
M3B	<0.001	10.3	10.3	0.910 (<0.001)	0.951 (<0.001)	0.003	6.226	6.229	0.902 (<0.001)	0.952 (<0.001)	
M4	<0.001	10.2	10.2	0.911 (<0.001)	0.952 (<0.001)	0.025	6.280	6.305	0.901 (<0.001)	0.953 (<0.001)	
M5	0.002	11.0	11.0	0.905 (<0.001)	0.951 (<0.001)	0.029	6.707	6.736	0.895 (<0.001)	0.954 (<0.001)	
M6	0.001	11.0	11.0	0.904 (<0.001)	0.952 (<0.001)	0.008	6.517	6.525	0.898 (<0.001)	0.953 (<0.001)	
M7A	<0.001	10.9	10.9	0.905 (<0.001)	0.952 (<0.001)	0.002	6.570	6.572	0.897 (<0.001)	0.954 (<0.001)	
M7B	<0.001	11.0	11.0	0.904 (<0.001)	0.951 (<0.001)	0.004	6.910	6.914	0.892 (<0.001)	0.943 (<0.001)	
M8A	0.001	10.1	10.1	0.912 (<0.001)	0.952 (<0.001)	0.003	6.072	6.075	0.905 (<0.001)	0.954 (<0.001)	
M8B	0.001	11.0	11.0	0.910 (<0.001)	0.952 (<0.001)	0.010	6.088	6.098	0.903 (<0.001)	0.953 (<0.001)	

a M0 = daily milk yield (DMY) estimated by doubling morning (AM) or evening (PM) milk yield; M1 = additive correction factor (ACF) model with categorical milking interval classes (MIC) and lactation months; M2A = ACF, model with continuous variables for milking interval and days in milk (DIM); M2B = M2A with ACF, computed on discretized MIC; M3A = linear regression of daily milk yield on milking interval and DIM; M3B = M3A with ACF, computed on discretized MIC; M4 = M3A with quadratic terms for milking interval and DIM; M5 = multiplicative correction factor (MCF) model according to Shook et al. (1980); M6 = MCF model according to DeLorenzo and Wiggans (1986); M7A = linear regression of AM or PM and proportion of DMY, on milking interval and DIM (Wiggans, 1986); M7B = M7A with MCF, computed for discretized MIC (Wiggans, 1986); M8A = exponential regression model (Wu et al., 2022); M8B = M8A with MCF, computed on discretized MIC.

b Var = variance; Bias^2^ = squared bias; MSE, mean squared error; Acc = *R* ^2^ accuracy; Cor = correlation between the estimated and actual DMY.

c Numbers in the brackets were standard errors of the *R* ^2^ accuracy estimates.

The standard deviation of the mean *R* ^2^ accuracy between the 30 CV replicates was 0 for M0 and less than 0.001 for all the remaining methods. Exactly, the standard deviation of *R* ^2^ accuracies between cross-validation replicates ranged between 0.00002 and 0.0001 for these methods in Holstein cows and between 0.0001 and 0.0005 in Jersey cows. By this definition, the *R* ^2^ accuracy is viewed as a population parameter. Based on paired *t*-test, we showed that the exponential regression model had highly significant mean *R* ^2^ accuracy than each of the existing methods (Holsteins: t = 584.8–37281; *p* < 2.2–16; Jerseys: 1178.5–5861.4; *p* < 2.2e-16). The model M0 had the lowest *R* ^2^ accuracy (0.821 in Holstein cows and 0.798 in Jersey cows). Compared to model M0, the ACF and MCF models, including the linear regression models, highly significantly improved the accuracies for estimating DMY (Holsteins: t = 8658.1–37281; *p* < 2.2e-16; Jerseys: 11.67–5861.4; *p* < 1.8e-12). The MCF and linear regression models had slightly higher accuracies of DMY estimates (0.904–0.912 in Holstein cows and 0.892–0.905 in Jersey cows) than the ACF models (0.902–0.910 in Holstein cows and 0.892–0.904 in Jersey cows). The exponential regression models, M8A and M8B, had the greatest *R* ^2^ accuracies of DMY estimates (0.910–0.912 in Holstein cows and 0.903–0.905 in Jersey cows). Based on a similar criterion, Liu et al. (2000) reported slightly higher *R* ^2^ accuracies (0.885) for doubling AM or PM approach in the German Holstein cows than ours in the US Holstein cows (0.821). The accuracies of estimated DMY (0.902–0.912) in the US Holstein cows that we obtained using the DeLorenzo and Wiggans (1986) model were within the accuracy range (0.900–0.914) in German Holstein cows obtained by Liu et al. (2000) using the same model. In addition to the genetic differences between German and the US Holstein cows, the accuracies of estimated DMY can vary with evaluation methods. Liu et al. (2000) employed in-sample evaluation, whereas we evaluated the accuracies by 10-fold cross-validation. As mentioned earlier, the accuracy obtained from cross-validation tends to be lower than that from in-sample evaluation because the former evaluations are prone to overfitting (Harkins and Douglas, 2004). Thus, comparing various methods is valid only when applied to the same dataset with the same evaluation strategy.

Correlation has been widely used to measure prediction accuracy, e.g., in genomic prediction and machine learning. However, correlation is not as informative as the *R* ^2^ accuracy for evaluating the performance of various models to estimate DMY. In the present study, all the models had similarly high correlations (0.951–0.952 in Holstein cows and 0.952–0.954 in Jersey cows) between the estimated and actual DMY, except that the model M0 had significantly lower corrections (0.927 in Holstein cows and 0.948 in Jersey cows). The standard deviation (i.e., standard error) of correlations between cross-validation replicates were all less than 0.0005. In statistics, correlation measures the degree of dependence between two random variables. Yet, correlation is not a precise measure of accuracy for two evident reasons. First, a correlation can be negative, but a valid accuracy measure is non-negative. Second and more importantly, a correlation does not account for estimation biases, meaning that two methods having identical corrections can vary drastically in the biases of the estimates. Hence, we recommend using the *R* ^2^ accuracy, instead of correction, as the measure of accuracy for estimating DMY.

A couple of reasons are worth noting for the lower accuracies with the ACF models than linear regression models. First, an ACF model is equivalent to assuming a fixed regression coefficient for partial milk yield, which can limit its predictability. For example, consider the models M2A and M2B. With some re-arrangements, these two models can be re-arranged into linear regression models of DMY on milk interval and DIM, plus a variable for AM or PM milk yield with a fixed regression coefficient ( b=2.0 ). The re-arranged models have similar model settings for predictor variables as the linear regression models, M3A and M3B, except that the linear models treat regression coefficients as unknown and estimated from the data. Possibly, by relaxing the restriction b=2.0 and estimating it from the data, the linear regression models (M3A and M3B) predicted the data better than the ACF models (M2A and M2B). Second, specific to ACF models with discrete regression variables (e.g., M1), it was challenged by data missing or insufficient data for some MIC, which led to a loss of accuracy for estimating DMY. In reality, deriving ACF from a regression model with discrete variables is also challenged as the number of categorical variables increases. Hence, the computation can be highly intensive or even not practically operational. For example, 20 MIC, 4 herd location regions, 4 years, 4 seasons, and 2 parities were considered. Then, there would be 20×4×4×4×2=2,560 specific classes for which ACF needed to be estimated if considering all these categorical variables at the same time.

Concerning an ACF or MCF model with continuous variables for milking intervals and DIM, discretizing a continuous variable to a categorical variable often leads to loss of information (and, therefore, accuracy) to some extent. Wu et al. (2022) showed analytically that computing ACF and MCF on discretized MIC led to a loss of accuracy of DMY estimates. This phenomenon was empirically observed in the Holstein and Jersey cows in the present study when comparing four pairs of models: M2A versus M2B, M3A versus M3B, M7A versus M7B, and M8A versus M8B. Each pair had the same model settings except that DMY were estimated with different strategies. The models labeled “A” (M2A, M3A, M7A, and M8A) estimated DMY directly based on estimated model parameters. Instead, the models labeled “B” (M2B, M3B, M7B, and M8B) computed ACF or MCF for discretized MICs after data fitting. Then, DMY were estimated through the calculated ACF or MCF. The models in group A consistently had smaller MSE and better accuracies than their counterparts in group B (Table 3). These results were an indication that discretizing milking interval time led to a loss of accuracy in estimated DMY. Hence, computing ACF or MCF without accounting for the loss due to discretizing MIC may be suboptimal when the linearity holds.

**FIGURE 3:**
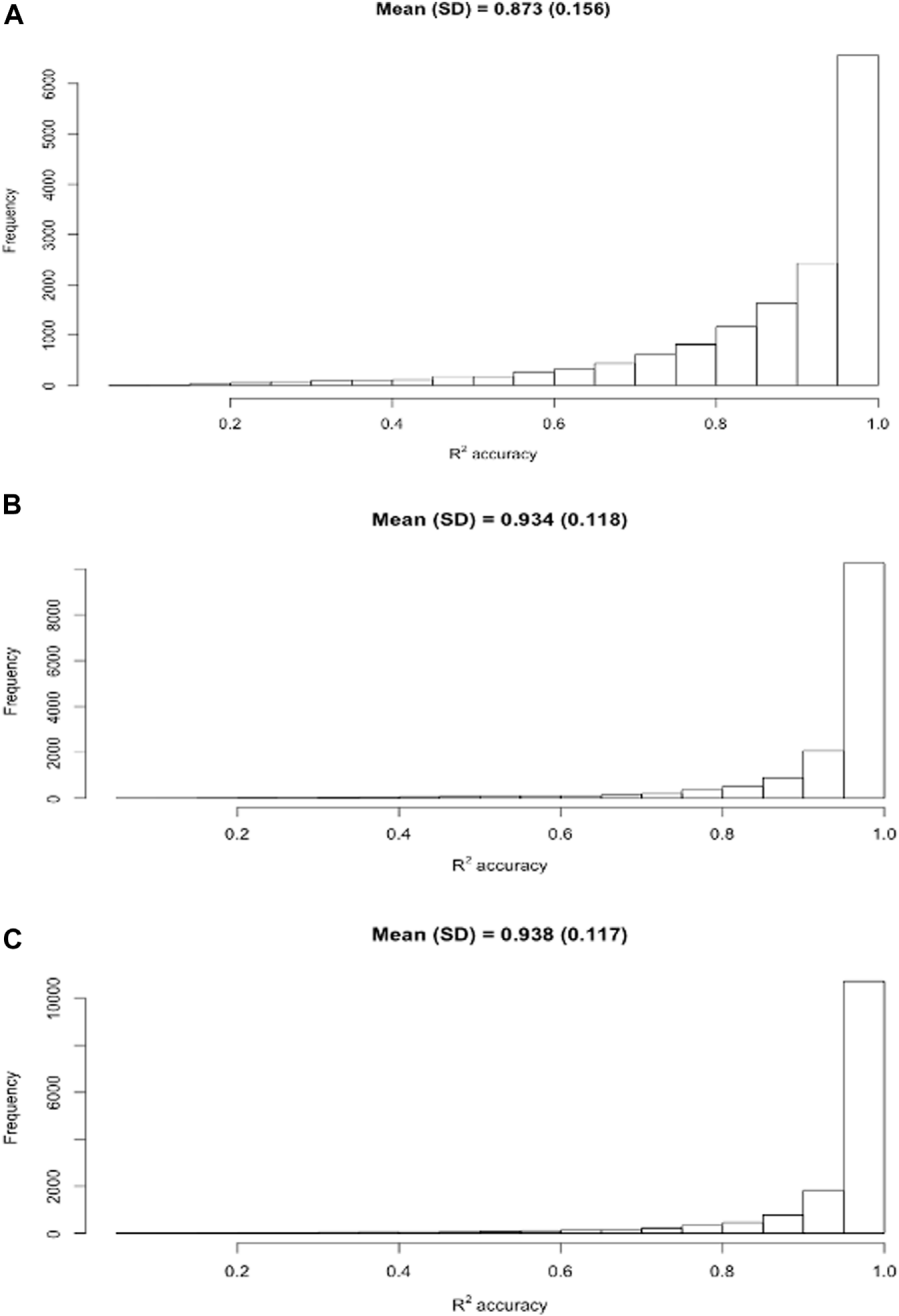
Distribution of individual *R* ^2^ accuracies of the estimated daily milk yield obtained using three models, M0 **(A)**, M2B **(B)**, and M7B **(C)**, respectively. M0 = two times AM or PM yield as the estimate of test-day milk yield; M2B = additive correction factor model implemented by regressing the difference between AM and PM yields on milking interval and days in milk; M7B = multiplicative correction factor model according to Wiggans (1986).

Relative to model M0 (doubling AM or PM milk yields), ACF and MCF models have considerably improved the DMY accuracy. To probe into the details, we computed the *R* ^2^ accuracies for individual cows based on three selected models, M0 (doubling AM or PM yields), one ACF model (M2B), and one MCF model (M7B). It came to our attention that mean individual *R* ^2^ accuracies were higher than the average *R* ^2^ accuracy population-wise across the 30 replicates. The distributions of individual *R* ^2^ accuracies in the Holstein cows obtained from these three models are shown in Figure 3. In particular, the distribution of individual R accuracies for the model M0 had a thicker tail than that for the model M2B or M7B. This was an indication that doubling AM or PM milk yields as the estimated DMY led to a higher percentage of the estimated DMY with lower accuracies, compared to the ACF and MCF models. The percentage of individual *R* ^2^ accuracies ≥ 0.90 were 59.6% (M0), 81.6% (M2B), and 83.4% (M7B). Average individual *R* ^2^ accuracy was 0.934 for M2B and 0.937 for M7B, respectively; both were substantially higher than the average individual *R* ^2^ accuracy (0.873) for M0. The medians of the *R* ^2^ accuracies were 0.927 for M0, 0.976 for M2B, and 0.980 for M7B, respectively, in the Holstein cows. The medians were consistently larger than the means. The MCF model (M7B) had a slightly higher mean *R* ^2^ accuracy than the ACF model (M2B). Unlike the standard deviations of the average *R* ^2^ accuracies between cross-validation replicates, which were all close to zero, the standard deviation of the individual *R* ^2^ accuracy was 0.135 for M0, 0.116 for M2B, and 0.115 for M7B. By Student’s *t*-test, the mean *R* ^2^ accuracies between M2B and M7B was not significantly different (t = 1.69, *p* = 0.091), yet they both were highly significantly greater than the mean *R* ^2^ accuracy of M0 (t = 29.4–31.1, *p* < 2.2e-16). Similar trends were observed in Jersey cows.

**FIGURE 4:**
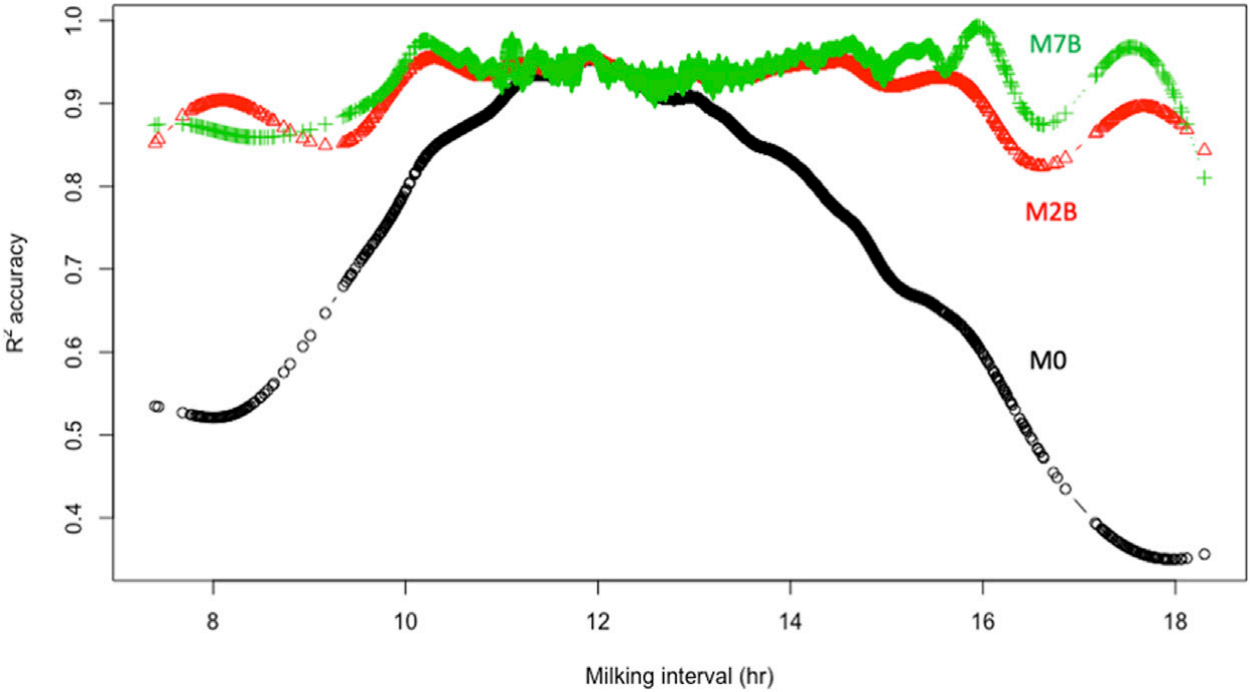
Relationships between smooth splines means of individual *R* ^2^ accuracies of the estimated daily milk yield and milking interval for three models, M0, M2B, and M7B. M0 = two times AM or PM yield as the estimate of test-day milk yield; M2B = additive correction factor model implemented by regressing the difference between AM and PM yields on milking interval and days in milk; M7B = multiplicative correction factor model according to Wiggans (1986).

Furthermore, the cubic smoothing spline (**CSS**) means of individual *R* ^2^ accuracies obtained from the three models were plotted against milking interval time in hours. (Figure 4). All three models had comparable means of individual *R* ^2^ accuracies when AM and PM milking intervals were approximately 12 h. Still, the average individual *R* ^2^ accuracy with the model M0 dropped drastically as the milking interval deviated from 12 h. The further it deviated from 12 h, the lower the average *R* ^2^ accuracy it had. In contrast, average individual *R* ^2^ accuracies for models M2B and M7B remained consistently high for milking intervals between 10 h and 16 h. They dropped slightly outside that range due to insufficient milking data. Hence, doubling AM or PM yield is equivalent to assuming a fixed multiplicative factor of 2.0 for AM and PM milk yields. It is valid (or approximately so) only for equal (12–12 h) AM and PM milking intervals but subject to large errors with unequal AM and PM milking intervals. Instead, ACF and MCF effectively provided adjustments to unequal milking intervals, leading to substantially improved DMY accuracies.

### Comparing Model Parameters

Model parameters were estimated and compared for four selected models (M2A, M3A, M7A, and M8A) using all milking data in Holstein and Jersey cows; each was implemented for AM or PM milkings separately and jointly (Table 4). The first two models, M2A and M3A, are the baseline models for the ACF models M2B and M3B. Both models (M2A and M3A) were implemented similarly yet with slightly different modeling assumptions. The model M2A equivalently assumed a fixed regression coefficient “2.0” for AM or PM milk yields, whereas the model M3A estimated the regression coefficient for AM or PM yield from the data. For example, the estimated regression coefficient with model M3A was 1.749 in Holstein cows and 1.750 in Jersey cows when AM and PM milk yields were analyzed jointly. Hence, the model M2A provided additive adjustments to two times AM or PM milk yields as the DMY estimates, whereas the model M3A provided additive adjustments to approximately 1.75 times AM or PM milk yields as the estimated DMY. Owing to this difference, other model parameters varied between both models. Overall, the model M3A had a slightly larger intercept than the model M2A in both datasets. The regression coefficients for milking intervals were all negative for both models. The absolute value of the regression coefficient for milking interval in the model M2A was larger than that in the model M3A. The model M3A would coincide precisely with the ACF model M2A if we could fix the regression coefficient for AM or PM milk yield to be 2.0 in the model M3A.

**TABLE 4:** Estimated parameters obtained from four models (M2A, M3A, M7A, and M8A), each implemented separately or jointly for known morning (AM) or evening (PM) milk yields ^a^ ^,^ ^b^.

Statistical model	Model parameter	Holstein			Jersey		
		AM	PM	Joint	AM	PM	Joint
M2A	$\alpha_{1}$	25.80 (0.431)	---	26.04 (0.302)	9.593 (1.170)	---	9.789 (0.807)
	$\alpha_{2}$	---	27.01 (0.870)	26.79 (0.285)	---	11.84 (0.951)	11.64 (0.692)
	β	−2.190 (0.035)	−2.222 (0.034)	−2.206 (0.024)	−0.898 (0.090)	−0.905 (0.085)	−0.889 (0.062)
	γ	0.001 (3E-4)	−0.001 (3E-4)	-4.7E-5 (2E-4)	0.001 (0.001)	-0.001 (0.001)	-1.4E-4 (4E-04)
M3A	$\alpha_{1}$	27.76 (0.404)	---	26.64 (0.283)	13.52 (1.402)	---	11.22 (0.701)
	$\alpha_{2}$	---	28.02 (0.382)	27.35 (0.267)	---	12.49 (0.947)	12.90 (0.652)
	β	−1.898 (0.033)	−1.934 (0.034)	−1.909 (0.024)	−0.797 (0.078)	−0.782 (0.086)	−0.746 (0.059)
	γ	−0.005 (3E-4)	−0.005 (3E-4)	−0.005 (2E-4)	−0.003 (0.001)	−0.003 (0.001)	−0.003 (0.001)
	b	1.720 (0.008)	1.780 (0.008)	1.749 (0.005)	1.664 (0.017)	1.860 (0.022)	1.750 (0.014)
M7A	$\alpha_{1}$	0.071 (0.008)	---	0.068 (0.005)	0.269 (0.029)	---	0.268 (0.020)
	$\alpha_{2}$	---	0.053 (0.007)	0.056 (0.005)	---	0.231 (0.024)	0.231 (0.017)
	β	0.036 (0.001)	0.037 (0.001)	0.037 (4E-04)	0.021 (0.002)	0.021 (0.002)	0.021 (0.002)
	γ	7E-06 (5E-06)	-5E-06 (5E-06)	8E-07 (4E-06)	2E-05 (1E-05)	-2E-05 (1E-05)	3.3E-06 (1E-05)
M8A	$\alpha_{1}$	1.779 (0018)	---	1.856 (0.013)	1.580 (0.067)	---	1.575 (0.048)
	$\alpha_{2}$	---	1.946 (0.017)	1.877 (0.012)	---	1.621 (0.060)	1.638 (0.042)
	β	−0.059 (0.001)	−0.070 (0.001)	−0.065 (0.001)	−0.037 (0.005)	−0.025 (0.005)	−0.032 (0.004)
	γ	-2E-04 (1E-05)	-2E-04 (1E-05)	-2E-04 (9E-06)	-3E-04 (3E-05)	-3E-04 (4E-05)	-3E-04 (3E-05)
	b	0.861 (0.004)	0.852 (0.004)	0.856 (0.003)	0.812 (0.010)	0.757 (0.011)	0.784 (0.008)

a M2A = additive correction factor model with continuous variables for milking interval and days in milk (DIM); M3A = linear regression of daily milk yield (DMY) on milking interval and DIM; M7A = linear regression of AM or PM and proportion of DMY, on milking interval and DIM (Wiggans, 1986); M8A = exponential regression model (Wu et al., 2022).

b $\alpha_{1}$ = intercepts for AM milk yield; $\alpha_{2}$ = intercept for PM milk yield; β = common regression coefficient for milking interval; γ = common regression coefficient for DIM; b = common regression coefficient for AM (or PM) milk yield (M3A) or the logarithm of AM or PM milk yield (M8A).

The models M7A and M8A are the baseline models for the MCF models, M7B and M8B. The MCF models represented substantially different modeling strategies (Table 2). For example, the former model (M7A) fitted AM or PM proportion of DMY as a linear function of milking interval and days in milk (Wiggans, 1986). In contrast, the latter (M8A) was an exponential regression model (Wu et al., 2022). We show that the model M8A was equivalent to a linear regression of the logarithm DMY on milking interval, days in milk, and the logarithm AM (or PM) milk yields through reparameterization. When AM and PM milk yields were analyzed jointly, the regression coefficient for milking interval was positive (0.037 in Holstein cows and 0.021 in Jersey cows) in the model M7A, whereas it was negative (0.065 in Holstein cows and 0.032 in Jersey cows) in the model M8A. The regression coefficient for the logarithm AM (or PM) milk yield was less than 1.0 (0.856 in Holstein cows and 0.784 in Jersey cows) in the model M8A.

**FIGURE 5:**
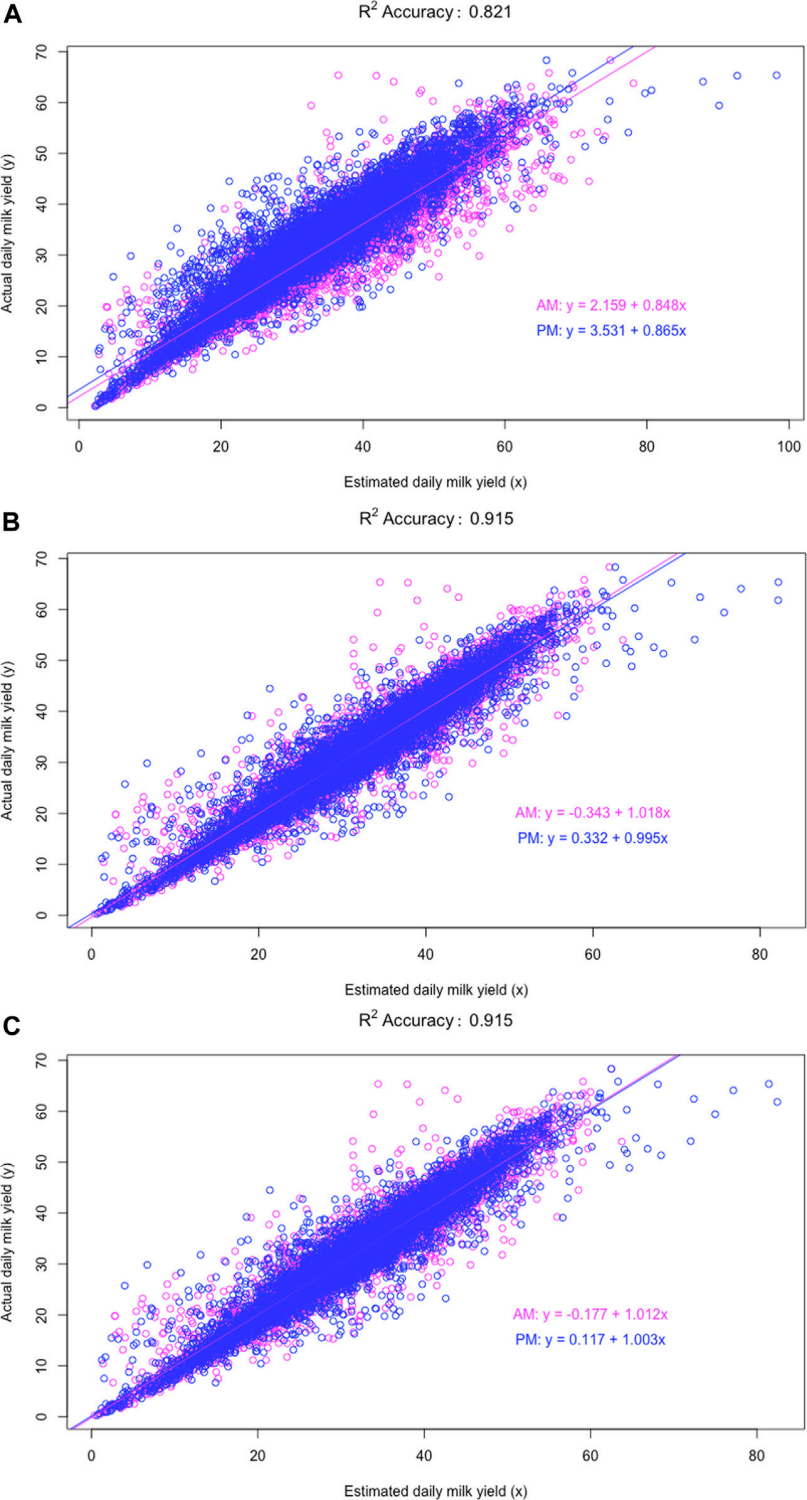
Scatterplot and linear regression fits of the actual daily milk yield against estimated daily milk yields under three scenarios: **(A)** estimating daily milk yield (DMY) by doubling morning (AM) or evening (PM) milk yields (model M0); **(B)** estimating DMY for known morning (AM) and evening (PM) milkings separately using the exponential regression model (model M8A; separate analysis); **(C)** estimating DMY for known AM and PM milkings jointly using the exponential regression model (model M8A; joint analysis).

Analyzing AM and PM milk yields separately led to slightly different model parameters in Holstein and Jersey cows (Table 4). Overall, the joint model had a smaller standard deviation of model parameters because the size of the data used to estimate these parameters doubled. Therefore, the joint analysis improved the precision of estimated model parameters by pooling AM and PM milk yields. Nevertheless, the accuracies of estimated DMY from separate analyses for AM or PM milkings increased only slightly compared to the joint analyses. Plots of actual and estimated DMY for the exponential regression model (M8A), implemented separately or jointly for AM and PM milkings, are shown in Figure 5B,C compared to the mode M0 (Figure 5A). The plots showed slight stratification between AM and PM milkings, which explained why separate analyses had better, although slightly, linear regression fits between the actual and estimated DMY than joint analyses. For the model M8A, separate analyses had smaller intercepts and the regression coefficient was closer to 1, indicating improved accuracies with the separate analyses. However, the extent of improved accuracies was very slight. The *R* ^2^ accuracy was 0.9151 for the separate accounting and 0.9147 for the joint analysis; both rounded to 0.915. Here, we show that the accuracy obtained from the in-sample evaluation was higher than that (0.912) from the 10-fold cross-validation. Similarly, the *R* ^2^ accuracies from separate analyses were almost identical to or slightly better than joint analyses for the other models. For example, the *R* ^2^ accuracies were 0.9040 with the joint analysis and 0.9042 with the separate analysis for the model M2A, 0.9128 (joint) and 0.9131 (separate) for the model M3A, and 0.9062 (joint) and 0.9063 (separate) for the model M7A. The differences in the *R* ^2^ accuracies were seen only in the third or fourth decimal points. Compared to model M8A, model M0 had considerably larger intercepts and the regression coefficients deviated substantially from 1.0. In other words, the model M8A has improved the DMY accuracies substantially compared to model M0. Similar conclusions hold all the ACF and MCF models and linear regression models, compared to doubling AM or PM yield as the daily yields.

**FIGURE 6:**
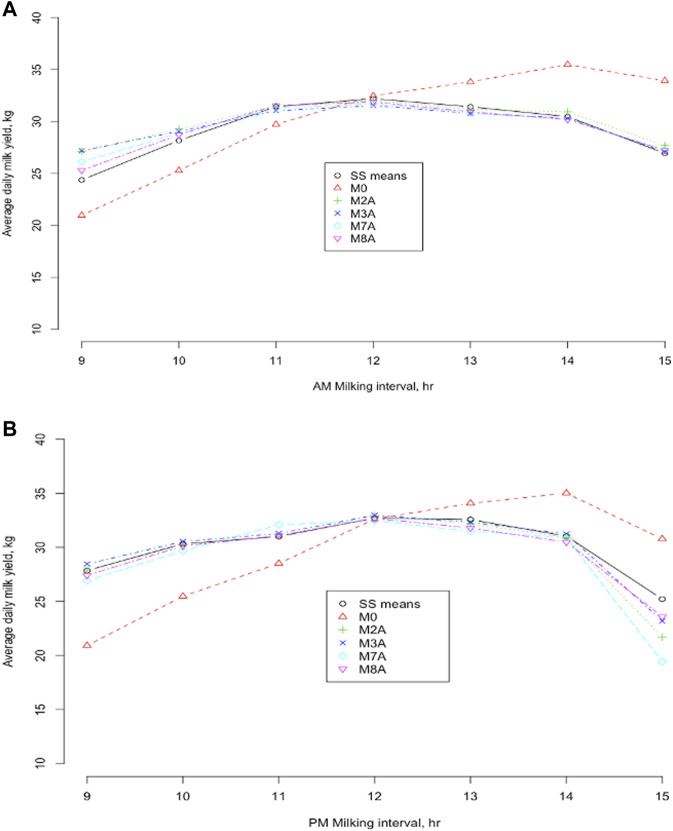
Average daily milk yields were obtained from five models and smooth spline (SS) means of the daily milk yield against morning **(A)** and evening **(B)** milking intervals from 9 to 15 h, respectively. M0 = daily milk yield (DMY) estimated as two times AM or PM yield; M2A = linear regression of the difference between morning (AM) and evening (PM) milk yields on milking interval and days in milk (DIM); M3A = linear regression of DMY on the milking interval and DIM; M7A = linear regression of AM or PM proportion of DMY on milking interval and DIM (Wiggans, 1986); M8A = exponential regression model (Wu et al., 2022).

Average DMY by milking intervals between 9 and 15 h were computed based on the estimated model parameters by joint analyses for the four selected models (M2A, M3A, M7A, and M8A), compared to the model M0 and the CSS means of actual DMY over milking interval (Figure 6). All the methods gave an average DMY comparable to the CSS means when AM and PM milking intervals were equal (12–12 h for AM and PM milking intervals). Still, they showed larger deviations with unequal AM and PM milkings. The model M0 had the largest deviations from the CSS means of DMY. Overall, the model M0 underestimated DMY with milking interval <12 h and overestimated DMY with milking interval >12 h. The more the AM (PM) milking interval departed from 12 h, the larger its deviation from the actual DMY. For the model M0, the average absolute deviation from the SCC means was 3.23 kg in Holstein and Jersey cows. Nevertheless, the deviations were much smaller for the ACF models (M2A and M3A) and the MCF models (M7A and M8A). The exponential regression model M8A had the smallest average absolute deviations from the CSS means of DMY (0.543 kg in the Holstein cows and 0.598 in the Jersey cows). For the other models M2A, M3A, and M7A, the average absolute deviation from the CSS mean varied from 0.568 (M3A) to 0.773 (M2A) in the Holstein cows and from 0.649 (M3A) to 0.914 (M2A) in the Jersey cows. These results also showed that the relation between the smoothed average DMY and milking interval time from 9 h to 15 h was not precisely linear (Figure 6). Early studies showed that DMY (including fat and solid-not-fat) were not linear with intervals beyond 12 h (Ragsdale et al., 1924; Bailey et al., 1955; Elliott and Brumby, 1955; Schmidt, 1960). In particular, Atashi and Hostens (2021) showed that milk and component productions, in relation to the interval between the current milking and the previous milking, showed an exponential increase at the beginning and later leveled off to an asymptote. This exponential behavior for milk production was assumed to be the result of cell degradation and milk present in the udder (Neal and Thornley, 1983).

### Comparing Additive and Multiplicative Correction Factors

**FIGURE 7:**
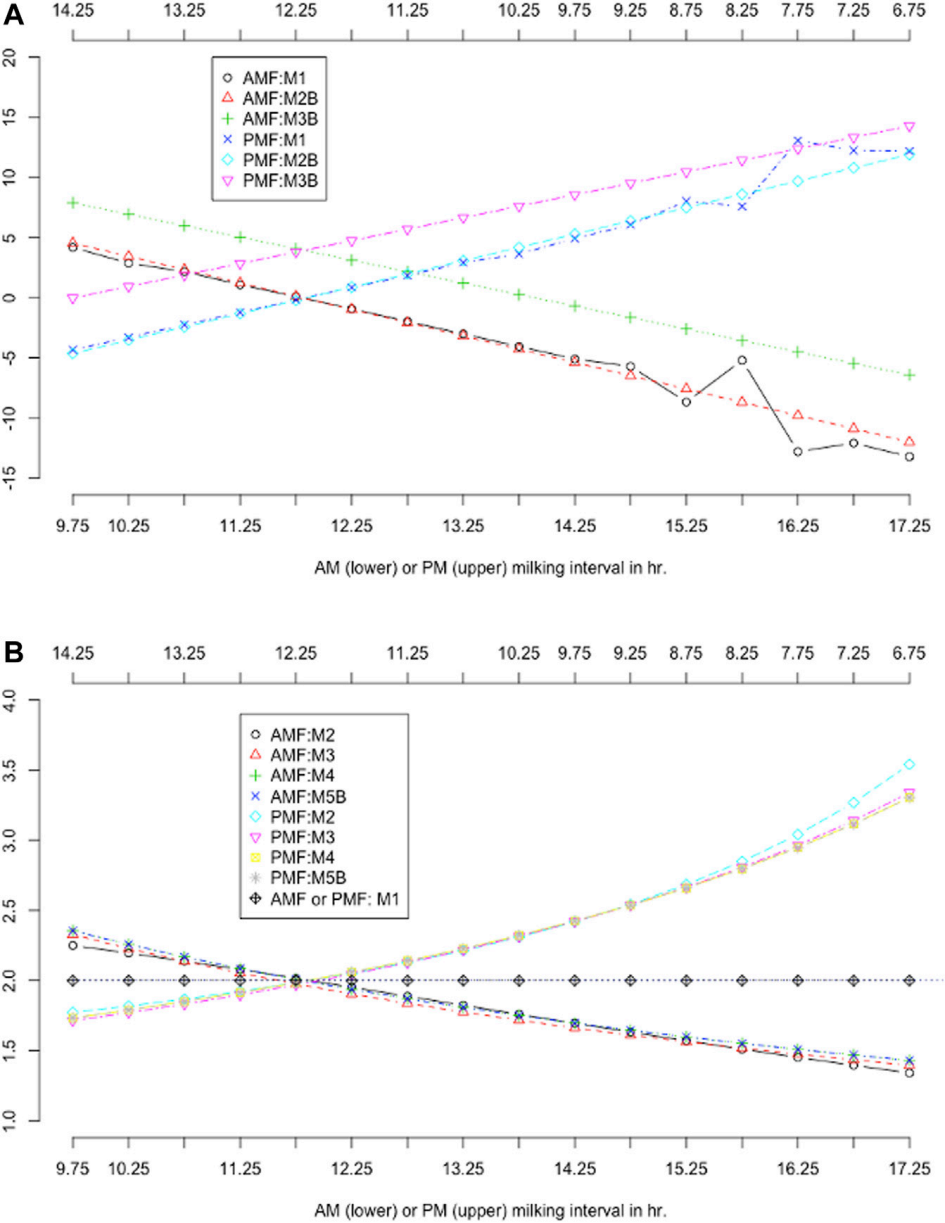
Comparison of additive correction factors **(A)** and multiplicative correction factors **(B)** obtained using different models. AMF = morning milk yield correction factors; PMF = evening milk yield correction factors. M0 = daily milk yield (DMY) estimated as two times AM or PM yield; M1 = additive correction factors (ACF) model with categorical milking interval (MIC) and lactation months; M2B = ACF model with continuous milking interval and days in milk (DIM); M3B = linear regression of DMY on milking interval and DIM, with ACFs computed for discretized MIC; M5 = multiplicative correction factor (MCF) model according to Shook et al. (1980); M6 = MCF model according to DeLorenzo and Wiggans (1986); M7B = MCF model according to Wiggans (1986); M8B = MCF model based on the exponential regression model (Wu et al., 2022).

Additive and multiplicative factors were computed based on the parameter values of the data density functions or smoothing functions. Plots of ACF and MCF by MIC are shown in Figure 7. The ACF models were implemented with slightly different model assumptions, yet they resulted in drastically different ACF values in two groups (Figure 7A). The two classic ACF models, M1 and M2B, equivalently assumed a fixed regression coefficient of 2.0 for AM or PM milk yield. Hence, both models gave roughly comparable ACF per MIC, except that ACF from M2B were smoothed, but those from M1 were not (Figure 7A). The M1 model had considerably large fluctuations of ACF when the milking interval was less than 9 h or greater than 15 h due to insufficient milking records. Instead, the model M2B fitted the data on a continuous variable for milking interval, and ACF were computed on discretized MIC regardless of the data size for a specific MIC. Hence, the model M2B was robust to insufficient milking records per MIC, provided that the data are sufficient in general. Within MIC, the sum of AM and PM ACF for each model was close to zero (which ranged −0.031 with M1 to −0.108 with M2B). The ACF computed from the linear regression model (M3B) were considerably larger than those based on the two ACF models (M1 and M2B). This was because the estimated regression coefficients (approximately 1.75; Table 4) from the linear regression models were less than the fixed regression coefficients (2.0) assumed in the ACF model. Hence, the classic ACF models provided additive adjustments to two times AM or PM milk yields as the estimated DMY. Still, the linear regression models provided additive adjustments to approximately 1.75 times AM or PM milk yields. Because of this difference, the ACF from the linear regression model should be larger than those from the ACF models. The sum of AM and PM ACF within MIC was greater than zero (i.e., 8.24 kg) for the model M3B, with the average ACF being 4.11 kg, in the Holstein cows. The average ACF from the linear regression model can be verified as follows. In the Holstein cows, the average AM and PM milk yields were 16.4 and 15.3 kg, respectively. The regression coefficients for AM and PM milk yields by the separate analyses were 1.72 and 1.78, respectively. Hence, the average difference in ACF between the linear regression model and the ACF model was approximately estimated to be

(2.0−1.72) ∗ 16.4+(2.0−1.78) ∗ 15.3 ≈4.0.

With equal (12–12 h) AM and PM milking intervals, the ACF obtained from the M1 and M2B models were all close to zero (0.09–0.123 kg in Holstein cows and -0.67–0.41 kg in Jersey cows). Because these two models each assumed a fixed regression coefficient of 2.0 for the AM or PM milk yield, we concluded that doubling AM or PM milk yields provided an approximate estimate of DMY with equal AM and PM milking intervals. Put in another way. With equal AM and PM milking intervals, the additive correction amount was zero beyond two-time AM or PM milk yield as the estimated DMY. The results agreed with some early studies. For example, Everet and Wadell (1970b) showed that the mean AM excluding PM milk production was -0.51–0.19 kg in Holstein cows and −0.35–0.27 kg in Jersey cows with approximately equal AM and PM milking intervals (720–749 min). In the present study, the average AM minus PM milk yield was 1.13 kg in Holstein cows and 1.75 kg in Jersey cows. Similarly, Everet and Wadell (1970b) reported that the average AM minus PM milk yield in Holstein cows was 1.28 kg in Holstein cows and 0.89 kg in Jersey cows. Both studies agreed with each other concerning the average AM minus PM milk yield, despite a 50-year gap. Nevertheless, the ranges of AM minus PM milk yield (and ACF) in our study were significantly larger than the ranges in Everet and Wadell (1970b) because daily milk production has increased considerably over the past decades.

Unlike the ACF model, the MCF models implemented substantially different modeling strategies (Table 2). Nevertheless, the computed MCF from various models all corresponded to ratios of daily-to-single milk yields, despite their statistical interpretations varied (Wu et al., 2022). Hence, MCF obtained using various methods were approximately comparable in the Holstein cows (Figure 7B). MCF agreed well between the four MCF models given AM milking between 11 and 15 h, or PM milking between 9 and 13 h. Yet, large differences were observed out of this range. MCF were approximately 2.0 when AM and PM milking intervals were both 12 h. The AM MCF was greater than 2.0 when the AM milking interval was less than 12, and it was less than 2.0 when the AM milking interval was greater than 12 h. A precisely opposite trend was observed with the PM MCF. These results again suggested that two times AM or PM milk yield was an approximate estimate of DMY with equal AM and PM milking intervals. Still, such approximation did not hold with uneven AM and PM milking intervals. Similar results were observed in Jersey cows as well.

## Conclusion

Estimated milk yields by doubling AM or PM milk yields were taken approximately assuming equal AM and PM milking intervals, but they were subject to large errors when AM and PM milking intervals were unequal. The more deviations of AM and PM milking intervals from 12–12 h, the larger errors it generated. ACF and MCF provided effective adjustments to the estimated DMY with unequal AM and PM milkings. ACF provided additive adjustments, evaluated by the expected difference between AM and PM milk yield for each MIC and other categorical variables when applicable. An ACF model equivalently assumed a fixed multiplier (2.0) for AM or PM milk yields. In reality, ACF models with many discrete variables are challenged by insufficient or missing data points for specific MIC categories. Similarly, a linear regression model was implemented as an ACF model which nevertheless estimated the multiplier (regression coefficient) for AM or PM milk yield from the data. Relaxing the limitation on the fixed multiplier for AM and PM milkings allowed linear regression models to fit and predict the data better than ACF models. Multiplicative correction factors were computed by ratios of daily yield to yield from a single milking. Thus, multiplying a known AM or PM yield by an MCF gave an estimated DMY. Overall, the MCF models outperformed the ACF models, providing more accurate DMY estimates in the Holstein and Jersey cows. Nevertheless, computed ACF or MCF on discretized milking interval time suffered from losing information, leading to larger errors and lower accuracies. The exponential regression model (Wu et al., 2022) had the smallest MSE and the greatest accuracies of DMY estimates. This new model is analogous to an exponential growth (or decay) function for DMY with the observed yield from single milking as the initial state and the change rate tuned by a linear function of milking interval and other variables when applicable. This exponential regression model provides a promising alternative tool for estimating DMY.

The present study represented a preliminary effort to revisit the existing statistical methods for estimating DMY, compared to the newly proposed exponential regression model, using milking data collected between 2006 and 2009. In a continuing effort, large-scaled high-resolution milking data are being collected for follow-up studies, jointly supported by the US Council on Dairy Cattle Breeding, the USDA Agricultural Genomics and Improvement Laboratories, and the National Dairy Herd Information Association. This is a 3-year data collection project. We expect that MCF in use will be updated by then. Finally, we illustrated the methods for estimating DMY in AM and PM milking plans. Yet, these methods and principles are generally applicable, either directly or with necessary modifications, to cows milked more than two times a day.

## Data Availability

The data analyzed in this study are subject to the following licenses/restrictions: The Holstein and Jersey milking record data maintained by CDCB are not publicly available, but can be requested to JD, subject to signing an official agreement for non-commercial use only. Requests to access these datasets should be directed to Joao Durr, Joao, durr@uscdcb.com.
